# 5-(4-Chloro­phen­yl)-3-(2-fur­yl)-1,2,4-triazolo[3,4-*a*]isoquinoline

**DOI:** 10.1107/S1600536810012924

**Published:** 2010-04-14

**Authors:** F. Nawaz Khan, P. Manivel, K. Prabakarana, Venkatesha R. Hathwar, Mehmet Akkurt

**Affiliations:** a Organic and Medicinal Chemistry Research Laboratory, Organic Chemistry Division, School of Advanced Sciences, VIT University, Vellore 632 014, Tamil Nadu, India; b Solid State and Structural Chemistry Unit, Indian Institute of Science, Bangalore 560 012, Karnataka, India; c Department of Physics, Faculty of Arts and Sciences, Erciyes University, 38039 Kayseri, Turkey

## Abstract

In the title mol­ecule, C_20_H_12_ClN_3_O, the triazoloisoquinoline ring system is nearly planar, with an r.m.s. deviation of 0.018 (3) Å and a maximum deviation of 0.034 (3) Å from the mean plane for the triazole ring C atom which is bonded to the benzene ring. The furan and benzene rings are twisted by 59.71 (14) and 66.95 (10)°, respectively, with respect to the mean plane of the triazoloisoquinoline ring system. The mol­ecular conformation is stabilized by an intra­molecular π–π inter­action [centroid-to-centroid distance = 3.5262 (18) Å]. The crystal packing is stabilized by weak C—H⋯π inter­actions and weak π–π inter­actions [centroid-to-centroid distance = 3.9431 (17) Å].

## Related literature

For a related crystal structure, see: Khan *et al.* (2010 ▶).
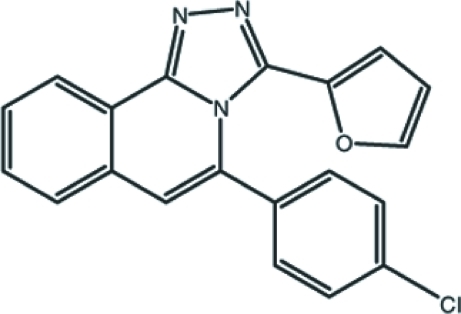


## Experimental

### 

#### Crystal data

C_20_H_12_ClN_3_O
*M* *_r_* = 345.78
Orthorhombic, 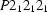

*a* = 9.0281 (9) Å
*b* = 12.6034 (11) Å
*c* = 14.6444 (15) Å
*V* = 1666.3 (3) Å^3^
*Z* = 4
Mo *K*α radiation
μ = 0.24 mm^−1^
*T* = 290 K
0.32 × 0.24 × 0.15 mm

#### Data collection

Oxford Xcalibur Eos (Nova) CCD detector diffractometer
Absorption correction: multi-scan (*CrysAlis PRO*; Oxford Diffraction, 2009 ▶) *T*_min_ = 0.933, *T*_max_ = 0.964
9280 measured reflections
3029 independent reflections
1831 reflections with *I* > 2σ(*I*)
*R*_int_ = 0.071

#### Refinement

*R*[*F* ^2^ > 2σ(*F* ^2^)] = 0.041
*wR*(*F* ^2^) = 0.082
*S* = 0.85
3029 reflections
227 parameters
H-atom parameters constrained
Δρ_max_ = 0.13 e Å^−3^
Δρ_min_ = −0.14 e Å^−3^
Absolute structure: Flack (1983 ▶), with 1245 Freidel pairs
Flack parameter: 0.00 (8)

### 

Data collection: *CrysAlis PRO* (Oxford Diffraction, 2009 ▶); cell refinement: *CrysAlis PRO*; data reduction: *CrysAlis PRO*; program(s) used to solve structure: *SHELXS97* (Sheldrick, 2008 ▶); program(s) used to refine structure: *SHELXL97* (Sheldrick, 2008 ▶); molecular graphics: *ORTEP-3* (Farrugia, 1997 ▶); software used to prepare material for publication: *WinGX* (Farrugia, 1999 ▶).

## Supplementary Material

Crystal structure: contains datablocks global, I. DOI: 10.1107/S1600536810012924/pv2272sup1.cif

Structure factors: contains datablocks I. DOI: 10.1107/S1600536810012924/pv2272Isup2.hkl

Additional supplementary materials:  crystallographic information; 3D view; checkCIF report

## Figures and Tables

**Table 1:** Hydrogen-bond geometry (Å, °) *Cg*2 is the centroid of the N1–N3/C1/C16 ring.

*D*—H⋯*A*	*D*—H	H⋯*A*	*D*⋯*A*	*D*—H⋯*A*
C20—H20⋯*Cg*2^i^	0.93	2.95	3.273 (4)	102

Symmetry code: (i) 
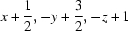
.

**Fig. 1.**
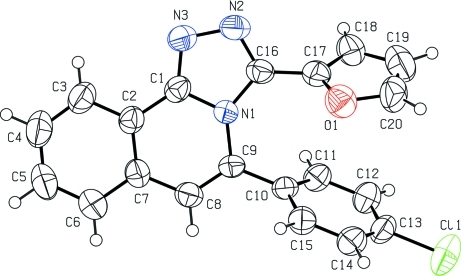
The title molecule with the atom numbering scheme. Displacement ellipsoids for non-H atoms are drawn at the 50% probability level.

**Fig. 2.**
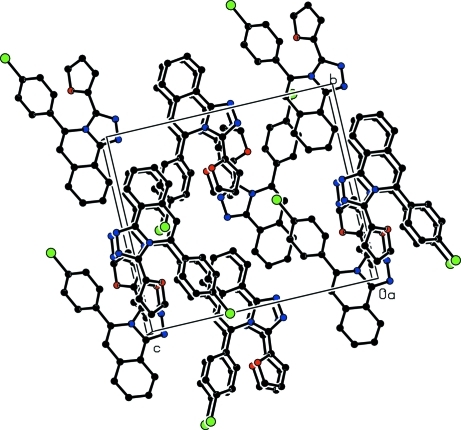
The packing diagram of (I) viewing down the a axis. H atoms have been omitted for clarity.

## supplementary crystallographic information

### Comment

As part of our search for new isoquinoline analogues (Khan *et al.*, 2010), we focused on synthesis of the title compound and its crystal structure is reported in this article.

In the title molecule (I), Fig. 1, the triazoloisoquinoline ring system (N1–N3/C1–C9/C16) is nearly planar, with an r.m.s. deviation of 0.018 (3) Å and a maximum deviation of 0.034 (3) Å from the mean plane for the triazole ring C16 atom which is bonded to the benzene ring. The furan (O1/C17–C20) and benzene (C10–C15) rings are twisted by 59.71 (14) and 66.95 (10)°, respectively, with respect to the mean plane of the triazoloisoquinoline ring system. The furan (O1/C17–C20) and benzene (C10–C15) rings make a dihedral angle of 21.76 (16)° with each other. The molecular conformation is stabilized by an intramolecular π–π interaction [Cg1···Cg5(x, y, z) = 3.5262 (18) Å; Cg1 and Cg5 are the centroids of the O1/C17–C20 and C10–C15 rings, respectively]. In the crystal structure, there is no classical hydrogen bonds. The crystal packing is stabilized by weak C—H···π interactions (Table 1) and weak π–π interactions [Cg1···Cg2(1/2 + x, 3/2 - y, 1 - z) = 3.9431 (17) Å; Cg2 is the centroid of the N1–N3/C1/C16 ring]. Fig. 2 shows the packing diagram of (I) viewing down the a axis.

### Experimental

2-(3-(4-Chlorophenylisoquinolin-1-yl)hydrazine (1 mmol) was condensed with furan-2-carbaldehyde (1.1 mmol) under refluxing conditions in isopropanol (10 ml) solvent to give the corresponding hydrazone in high yield. After removal of solvent the compound was then oxidatively cyclized in nitrobenzene (10 ml) at 473 K. The product was recrystallized from dichlomethane to give block-shaped crystals.

### Refinement

H atoms were placed in calculated positions with C—H = 0.93 Å and were included in the refinement in the riding model approximation, with *U_iso_*(H) = 1.2*U_eq_*(C).

### Crystal data

**Table:**

C_20_H_12_ClN_3_O	*F*(000) = 712
*M**_r_* = 345.78	*D*_x_ = 1.378 Mg m^−^^3^
Orthorhombic, *P*2_1_2_1_2_1_	Mo *K*α radiation, λ = 0.71073 Å
Hall symbol: P 2ac 2ab	Cell parameters from 1165 reflections
*a* = 9.0281 (9) Å	θ = 1.7–20.6°
*b* = 12.6034 (11) Å	µ = 0.24 mm^−^^1^
*c* = 14.6444 (15) Å	*T* = 290 K
*V* = 1666.3 (3) Å^3^	Block, colourless
*Z* = 4	0.32 × 0.24 × 0.15 mm

### Data collection

**Table:**

Oxford Xcalibur Eos (Nova) CCD detector diffractometer	3029 independent reflections
Radiation source: Enhance (Mo) X-ray Source	1831 reflections with *I* > 2σ(*I*)
graphite	*R*_int_ = 0.071
ω scans	θ_max_ = 25.5°, θ_min_ = 3.1°
Absorption correction: multi-scan (*CrysAlis PRO*; Oxford Diffraction, 2009)	*h* = −8→10
*T*_min_ = 0.933, *T*_max_ = 0.964	*k* = −14→15
9280 measured reflections	*l* = −17→17

### Refinement

**Table:**

Refinement on *F*^2^	Hydrogen site location: inferred from neighbouring sites
Least-squares matrix: full	H-atom parameters constrained
*R*[*F*^2^ > 2σ(*F*^2^)] = 0.041	*w* = 1/[σ^2^(*F*_o_^2^) + (0.0329*P*)^2^] where *P* = (*F*_o_^2^ + 2*F*_c_^2^)/3
*wR*(*F*^2^) = 0.082	(Δ/σ)_max_ = 0.001
*S* = 0.85	Δρ_max_ = 0.13 e Å^−^^3^
3029 reflections	Δρ_min_ = −0.14 e Å^−^^3^
227 parameters	Extinction correction: *SHELXL97* (Sheldrick, 2008), Fc^*^ = kFc[1+0.001Fc^2^λ^3^/sin(2θ)]^-1/4^
0 restraints	Extinction coefficient: 0.0109 (10)
Primary atom site location: structure-invariant direct methods	Absolute structure: Flack (1983), with 1245 Freidel pairs
Secondary atom site location: difference Fourier map	Flack parameter: 0.00 (8)

### Special details

**Table:**

Geometry. Bond distances, angles etc. have been calculated using the rounded fractional coordinates. All su's are estimated from the variances of the (full) variance-covariance matrix. The cell esds are taken into account in the estimation of distances, angles and torsion angles
Refinement. Refinement on *F*^2^ for ALL reflections except those flagged by the user for potential systematic errors. Weighted *R*-factors wR and all goodnesses of fit *S* are based on *F*^2^, conventional *R*-factors *R* are based on *F*, with *F* set to zero for negative *F*^2^. The observed criterion of *F*^2^ > σ(*F*^2^) is used only for calculating -*R*-factor-obs etc. and is not relevant to the choice of reflections for refinement. *R*-factors based on *F*^2^ are statistically about twice as large as those based on *F*, and *R*-factors based on ALL data will be even larger.

### Fractional atomic coordinates and isotropic or equivalent isotropic displacement parameters (Å^2^)

**Table:**

	*x*	*y*	*z*	*U*_iso_*/*U*_eq_	
Cl1	0.33125 (9)	1.00293 (7)	0.16049 (6)	0.0937 (3)	
O1	0.5357 (2)	0.77175 (16)	0.41053 (13)	0.0713 (8)	
N1	0.3343 (2)	0.56533 (14)	0.42785 (13)	0.0434 (7)	
N2	0.4295 (2)	0.58135 (18)	0.56552 (15)	0.0607 (9)	
N3	0.3791 (3)	0.47806 (17)	0.55611 (15)	0.0588 (8)	
C1	0.3237 (3)	0.46973 (19)	0.47266 (18)	0.0485 (9)	
C2	0.2614 (3)	0.3788 (2)	0.42840 (18)	0.0494 (9)	
C3	0.2546 (3)	0.2798 (2)	0.4715 (2)	0.0667 (11)	
C4	0.1926 (4)	0.1962 (2)	0.4261 (2)	0.0837 (14)	
C5	0.1397 (4)	0.2076 (3)	0.3387 (2)	0.0833 (16)	
C6	0.1482 (3)	0.3040 (2)	0.29456 (19)	0.0675 (11)	
C7	0.2094 (3)	0.39175 (19)	0.33964 (18)	0.0500 (10)	
C8	0.2207 (3)	0.4941 (2)	0.29680 (17)	0.0520 (9)	
C9	0.2803 (2)	0.57833 (19)	0.33756 (16)	0.0420 (8)	
C10	0.2922 (3)	0.68419 (19)	0.29424 (16)	0.0440 (9)	
C11	0.2102 (3)	0.7688 (2)	0.32553 (17)	0.0512 (10)	
C12	0.2214 (3)	0.8674 (2)	0.28522 (19)	0.0600 (11)	
C13	0.3148 (3)	0.8789 (2)	0.21205 (19)	0.0574 (10)	
C14	0.3970 (3)	0.7960 (2)	0.17858 (18)	0.0598 (11)	
C15	0.3843 (3)	0.6977 (2)	0.21980 (17)	0.0552 (10)	
C16	0.4029 (3)	0.6318 (2)	0.48920 (17)	0.0491 (9)	
C17	0.4389 (3)	0.7432 (2)	0.47754 (19)	0.0538 (10)	
C18	0.3987 (3)	0.8269 (2)	0.5256 (2)	0.0627 (11)	
C19	0.4689 (4)	0.9155 (3)	0.4879 (2)	0.0837 (14)	
C20	0.5505 (4)	0.8809 (3)	0.4193 (3)	0.0800 (14)	
H3	0.29170	0.27110	0.53030	0.0800*	
H4	0.18610	0.13060	0.45490	0.1000*	
H5	0.09780	0.14970	0.30900	0.1000*	
H6	0.11350	0.31090	0.23510	0.0810*	
H8	0.18460	0.50190	0.23770	0.0620*	
H11	0.14630	0.75940	0.37470	0.0610*	
H12	0.16690	0.92460	0.30710	0.0720*	
H14	0.46020	0.80580	0.12910	0.0720*	
H15	0.43810	0.64050	0.19730	0.0660*	
H18	0.33520	0.82690	0.57550	0.0750*	
H19	0.45980	0.98540	0.50740	0.1010*	
H20	0.60940	0.92350	0.38220	0.0960*	

### Atomic displacement parameters (Å^2^)

**Table:**

	*U*^11^	*U*^22^	*U*^33^	*U*^12^	*U*^13^	*U*^23^
Cl1	0.0975 (6)	0.0700 (5)	0.1137 (7)	−0.0069 (5)	0.0076 (5)	0.0403 (6)
O1	0.0635 (12)	0.0737 (15)	0.0768 (14)	−0.0078 (11)	0.0015 (12)	−0.0030 (13)
N1	0.0512 (13)	0.0416 (12)	0.0374 (11)	−0.0028 (11)	−0.0005 (11)	−0.0017 (11)
N2	0.0728 (15)	0.0637 (16)	0.0455 (14)	0.0032 (13)	−0.0107 (12)	−0.0025 (14)
N3	0.0732 (15)	0.0575 (16)	0.0457 (13)	0.0034 (12)	−0.0032 (12)	0.0029 (12)
C1	0.0557 (17)	0.0445 (16)	0.0453 (16)	0.0088 (13)	0.0062 (15)	0.0033 (14)
C2	0.0567 (17)	0.0442 (16)	0.0472 (16)	0.0006 (13)	0.0102 (14)	0.0012 (15)
C3	0.080 (2)	0.0570 (19)	0.0632 (19)	−0.0034 (15)	0.0079 (17)	0.0130 (18)
C4	0.109 (3)	0.054 (2)	0.088 (2)	−0.0159 (19)	0.010 (2)	0.010 (2)
C5	0.104 (3)	0.054 (2)	0.092 (3)	−0.0220 (19)	−0.002 (2)	−0.011 (2)
C6	0.076 (2)	0.060 (2)	0.0665 (19)	−0.0110 (17)	−0.0065 (17)	−0.0052 (18)
C7	0.0521 (17)	0.0433 (16)	0.0547 (18)	−0.0020 (13)	0.0042 (14)	−0.0043 (15)
C8	0.0570 (16)	0.0545 (17)	0.0444 (16)	0.0003 (14)	−0.0038 (13)	−0.0045 (15)
C9	0.0442 (14)	0.0477 (16)	0.0341 (14)	0.0027 (12)	0.0006 (12)	−0.0005 (14)
C10	0.0465 (15)	0.0498 (16)	0.0358 (14)	−0.0018 (13)	−0.0030 (13)	0.0016 (13)
C11	0.0491 (17)	0.0550 (17)	0.0495 (16)	0.0003 (13)	0.0083 (13)	0.0052 (15)
C12	0.0600 (18)	0.0530 (18)	0.067 (2)	0.0057 (14)	0.0012 (16)	0.0066 (16)
C13	0.0597 (18)	0.0538 (17)	0.0587 (18)	−0.0109 (16)	−0.0070 (16)	0.0193 (16)
C14	0.0611 (19)	0.067 (2)	0.0513 (17)	−0.0080 (15)	0.0113 (14)	0.0033 (17)
C15	0.0623 (17)	0.0530 (18)	0.0504 (16)	−0.0013 (14)	0.0093 (15)	−0.0030 (15)
C16	0.0546 (16)	0.0521 (17)	0.0407 (16)	0.0045 (14)	−0.0016 (13)	−0.0055 (15)
C17	0.0525 (17)	0.0586 (19)	0.0502 (18)	−0.0028 (15)	−0.0081 (15)	0.0001 (17)
C18	0.0672 (19)	0.0539 (18)	0.0670 (19)	0.0022 (16)	0.0031 (16)	−0.0094 (17)
C19	0.088 (2)	0.060 (2)	0.103 (3)	0.000 (2)	−0.018 (2)	−0.018 (2)
C20	0.073 (2)	0.064 (2)	0.103 (3)	−0.0245 (19)	−0.019 (2)	0.016 (2)

### Geometric parameters (Å, °)

**Table:**

Cl1—C13	1.742 (3)	C11—C12	1.380 (4)
O1—C17	1.362 (3)	C12—C13	1.371 (4)
O1—C20	1.388 (4)	C13—C14	1.372 (4)
N1—C1	1.375 (3)	C14—C15	1.383 (4)
N1—C9	1.419 (3)	C16—C17	1.451 (4)
N1—C16	1.376 (3)	C17—C18	1.319 (4)
N2—N3	1.386 (3)	C18—C19	1.398 (4)
N2—C16	1.308 (3)	C19—C20	1.320 (5)
N3—C1	1.325 (3)	C3—H3	0.9300
C1—C2	1.432 (4)	C4—H4	0.9300
C2—C3	1.400 (4)	C5—H5	0.9300
C2—C7	1.392 (4)	C6—H6	0.9300
C3—C4	1.366 (4)	C8—H8	0.9300
C4—C5	1.374 (4)	C11—H11	0.9300
C5—C6	1.378 (4)	C12—H12	0.9300
C6—C7	1.402 (4)	C14—H14	0.9300
C7—C8	1.438 (4)	C15—H15	0.9300
C8—C9	1.332 (3)	C18—H18	0.9300
C9—C10	1.481 (3)	C19—H19	0.9300
C10—C11	1.377 (4)	C20—H20	0.9300
C10—C15	1.382 (4)		
			
Cl1···C18^i^	3.579 (3)	C16···C11	3.428 (4)
Cl1···C16^ii^	3.634 (3)	C17···C11	3.053 (4)
Cl1···N1^ii^	3.378 (2)	C17···C10	3.084 (4)
Cl1···C9^ii^	3.634 (2)	C18···Cl1^viii^	3.579 (3)
Cl1···H18^i^	2.9000	C18···C11	3.467 (4)
O1···N1	3.184 (3)	C19···C1^v^	3.562 (5)
O1···C10	2.992 (3)	C20···C16^v^	3.456 (5)
O1···C11	3.192 (3)	C20···C12	3.565 (5)
O1···C15	3.247 (3)	C20···C1^v^	3.483 (5)
N1···O1	3.184 (3)	C2···H14^iii^	2.8100
N1···N2	2.201 (3)	C3···H14^iii^	2.9800
N1···Cl1^iii^	3.378 (2)	C8···H15	3.0600
N2···N1	2.201 (3)	C14···H3^vii^	2.8900
N3···N1	2.214 (3)	C15···H8	3.0700
N2···H6^iv^	2.8600	C17···H11	3.0500
N2···H11^v^	2.9400	C17···H11^v^	2.8600
N2···H12^v^	2.8400	C18···H11^v^	2.8800
N2···H8^iv^	2.9200	H3···N3	2.7500
N3···H3	2.7500	H3···C14^iv^	2.8900
N3···H8^iv^	2.7300	H6···H8	2.4900
N3···H20^vi^	2.8800	H6···N2^vii^	2.8600
C1···C19^vi^	3.562 (5)	H8···C15	3.0700
C1···C20^vi^	3.483 (5)	H8···H6	2.4900
C3···C14^iv^	3.462 (4)	H8···N2^vii^	2.9200
C9···Cl1^iii^	3.634 (2)	H8···N3^vii^	2.7300
C10···C17	3.084 (4)	H11···C17	3.0500
C10···O1	2.992 (3)	H11···N2^vi^	2.9400
C11···O1	3.192 (3)	H11···C17^vi^	2.8600
C11···C17	3.053 (4)	H11···C18^vi^	2.8800
C11···C18	3.467 (4)	H12···N2^vi^	2.8400
C11···C16	3.428 (4)	H14···C2^ii^	2.8100
C12···C20	3.565 (5)	H14···C3^ii^	2.9800
C14···C3^vii^	3.462 (4)	H15···C8	3.0600
C15···O1	3.247 (3)	H18···Cl1^viii^	2.9000
C16···Cl1^iii^	3.634 (3)	H20···N3^v^	2.8800
C16···C20^vi^	3.456 (5)		
			
C17—O1—C20	104.9 (2)	N1—C16—N2	110.2 (2)
C1—N1—C9	121.46 (19)	N1—C16—C17	127.8 (2)
C1—N1—C16	104.7 (2)	N2—C16—C17	122.0 (2)
C9—N1—C16	133.9 (2)	O1—C17—C16	118.9 (2)
N3—N2—C16	108.1 (2)	O1—C17—C18	110.5 (2)
N2—N3—C1	106.9 (2)	C16—C17—C18	130.5 (3)
N1—C1—N3	110.2 (2)	C17—C18—C19	107.7 (3)
N1—C1—C2	120.8 (2)	C18—C19—C20	106.9 (3)
N3—C1—C2	129.0 (2)	O1—C20—C19	110.1 (3)
C1—C2—C3	121.8 (2)	C2—C3—H3	120.00
C1—C2—C7	117.5 (2)	C4—C3—H3	120.00
C3—C2—C7	120.7 (2)	C3—C4—H4	119.00
C2—C3—C4	119.1 (3)	C5—C4—H4	120.00
C3—C4—C5	121.0 (3)	C4—C5—H5	120.00
C4—C5—C6	120.7 (3)	C6—C5—H5	120.00
C5—C6—C7	119.8 (3)	C5—C6—H6	120.00
C2—C7—C6	118.7 (2)	C7—C6—H6	120.00
C2—C7—C8	119.3 (2)	C7—C8—H8	118.00
C6—C7—C8	122.0 (2)	C9—C8—H8	118.00
C7—C8—C9	123.3 (2)	C10—C11—H11	120.00
N1—C9—C8	117.7 (2)	C12—C11—H11	119.00
N1—C9—C10	118.6 (2)	C11—C12—H12	121.00
C8—C9—C10	123.7 (2)	C13—C12—H12	121.00
C9—C10—C11	121.1 (2)	C13—C14—H14	121.00
C9—C10—C15	119.5 (2)	C15—C14—H14	121.00
C11—C10—C15	119.4 (2)	C10—C15—H15	120.00
C10—C11—C12	121.1 (2)	C14—C15—H15	120.00
C11—C12—C13	118.3 (2)	C17—C18—H18	126.00
Cl1—C13—C12	119.1 (2)	C19—C18—H18	126.00
Cl1—C13—C14	118.8 (2)	C18—C19—H19	127.00
C12—C13—C14	122.1 (2)	C20—C19—H19	127.00
C13—C14—C15	118.8 (2)	O1—C20—H20	125.00
C10—C15—C14	120.3 (2)	C19—C20—H20	125.00
			
C20—O1—C17—C16	−177.9 (3)	C3—C4—C5—C6	0.1 (5)
C20—O1—C17—C18	−1.2 (3)	C4—C5—C6—C7	−0.9 (5)
C17—O1—C20—C19	0.6 (4)	C5—C6—C7—C2	0.5 (4)
C9—N1—C1—N3	−178.9 (2)	C5—C6—C7—C8	−179.9 (3)
C9—N1—C1—C2	1.9 (4)	C2—C7—C8—C9	0.7 (4)
C16—N1—C1—N3	1.2 (3)	C6—C7—C8—C9	−179.0 (3)
C16—N1—C1—C2	−178.0 (2)	C7—C8—C9—N1	−0.3 (4)
C1—N1—C9—C8	−1.0 (3)	C7—C8—C9—C10	−179.8 (2)
C1—N1—C9—C10	178.6 (2)	N1—C9—C10—C11	−67.9 (3)
C16—N1—C9—C8	178.8 (3)	N1—C9—C10—C15	113.7 (3)
C16—N1—C9—C10	−1.6 (4)	C8—C9—C10—C11	111.7 (3)
C1—N1—C16—N2	−1.0 (3)	C8—C9—C10—C15	−66.7 (3)
C1—N1—C16—C17	−178.7 (3)	C9—C10—C11—C12	179.9 (2)
C9—N1—C16—N2	179.1 (2)	C15—C10—C11—C12	−1.7 (4)
C9—N1—C16—C17	1.4 (4)	C9—C10—C15—C14	−179.9 (2)
C16—N2—N3—C1	0.4 (3)	C11—C10—C15—C14	1.7 (4)
N3—N2—C16—N1	0.4 (3)	C10—C11—C12—C13	1.0 (4)
N3—N2—C16—C17	178.3 (2)	C11—C12—C13—Cl1	−179.7 (2)
N2—N3—C1—N1	−1.0 (3)	C11—C12—C13—C14	−0.3 (4)
N2—N3—C1—C2	178.2 (3)	Cl1—C13—C14—C15	179.7 (2)
N1—C1—C2—C3	177.6 (2)	C12—C13—C14—C15	0.4 (4)
N1—C1—C2—C7	−1.4 (4)	C13—C14—C15—C10	−1.1 (4)
N3—C1—C2—C3	−1.5 (5)	N1—C16—C17—O1	−62.7 (4)
N3—C1—C2—C7	179.5 (3)	N1—C16—C17—C18	121.5 (3)
C1—C2—C3—C4	179.4 (3)	N2—C16—C17—O1	119.9 (3)
C7—C2—C3—C4	−1.7 (4)	N2—C16—C17—C18	−56.0 (4)
C1—C2—C7—C6	179.8 (2)	O1—C17—C18—C19	1.4 (3)
C1—C2—C7—C8	0.2 (4)	C16—C17—C18—C19	177.5 (3)
C3—C2—C7—C6	0.8 (4)	C17—C18—C19—C20	−0.9 (4)
C3—C2—C7—C8	−178.8 (3)	C18—C19—C20—O1	0.2 (4)
C2—C3—C4—C5	1.2 (5)		

Symmetry codes: (i) −*x*+1/2, −*y*+2, *z*−1/2; (ii) −*x*+1, *y*+1/2, −*z*+1/2; (iii) −*x*+1, *y*−1/2, −*z*+1/2; (iv) −*x*+1/2, −*y*+1, *z*+1/2; (v) *x*+1/2, −*y*+3/2, −*z*+1; (vi) *x*−1/2, −*y*+3/2, −*z*+1; (vii) −*x*+1/2, −*y*+1, *z*−1/2; (viii) −*x*+1/2, −*y*+2, *z*+1/2.

### Hydrogen-bond geometry (Å, °)

**Table:**

*D*—H···*A*	*D*—H	H···*A*	*D*···*A*	*D*—H···*A*
C20—H20···Cg2^v^	0.93	2.95	3.273 (4)	102

Symmetry codes: (v) *x*+1/2, −*y*+3/2, −*z*+1.
